# Multi-metal-resistant Staphylococcus warneri strain TWSL_1: revealing heavy metal-resistant genomic features by whole-genome sequencing and analysis

**DOI:** 10.1099/acmi.0.000954.v5

**Published:** 2025-05-27

**Authors:** Dilani Chathurika Dissanayake, Naduviladath Vishvanath Chandrasekharan, Champika Dilrukshi Wijayarathna

**Affiliations:** 1 Department of Chemistry, Faculty of Science, University of Colombo, Colombo 03, Sri Lanka; 2 University of Alabama at Birmingham, Birmingham, Alabama, USA; 3 Sri Lanka Institute of Biotechnology, Thalagala road, Pitipana, Homagama, Sri Lanka

**Keywords:** genome sequence, heavy metal resistance, *Staphylococcus warneri*, TWSL_1, whole genome, phylogeny

## Abstract

This study explores the genomic basis of heavy metal resistance in *Staphylococcus warneri* strain TWSL_1, isolated from industrial textile effluent. The strain exhibited strong resistance to Cd²^+^, Pb²^+^ and Cu²^+^, with MICs of 50, 1,200 and 75 mg l^−1^, respectively. Whole-genome sequencing revealed a 2.66 Mb genome with 2,567 CDSs and a 99.81% average nucleotide identity to *S. warneri* WS479. Comparative genomic analysis at the genus level revealed that *S. warneri* strain TWSL_1 possesses a unique and expanded repertoire of heavy metal resistance genes, including the cadmium efflux system accessory protein and cadmium resistance protein, which are absent in pathogenic *Staphylococcus* sp. used for the comparison. Phylogenetic analysis confirmed its classification within *S. warneri*, with strong bootstrap support (100). Functional annotation highlighted metabolic versatility and stress response capabilities, supporting its adaptation to metal-rich environments. *S. warneri* TWSL_1 demonstrated high Pb²^+^ removal efficiency, reducing concentrations by over 70%. These findings highlight *S. warneri* TWSL_1 as a promising candidate for heavy metal bioremediation with potential applications in environmental detoxification and monitoring strategies.

**Impact Statement**

This study uncovers the genomic architecture underlying the exceptional heavy metal resistance of *Staphylococcus warneri* strain TWSL_1, isolated from industrial textile effluent. Using whole-genome sequencing, we identified key genetic determinants, including the cadmium efflux system and copper resistance proteins, critical for cadmium, lead and copper detoxification. These findings enhance our understanding of bacterial adaptation to metal stress and contribute valuable insights into environmental biotechnology. By demonstrating the strain’s strong bioremediation potential, this research highlights the practical application of TWSL_1 in mitigating heavy metal pollution from industrial sources such as textile dyeing and metal processing, offering eco-friendly solutions for environmental restoration.

## Data Summary

The complete genome sequence of *Staphylococcus warneri* strain TWSL_1 has been deposited in the NCBI GenBank database under accession number CP135051, providing open access to the assembled and annotated genome. The 16S rRNA gene sequence is available under accession number OR574394.1. All additional datasets used in this study including genome assemblies, annotations, and comparative analysis files are provided in the supplementary materials. These data support the genomic identification of key heavy metal resistance genes and allow for further exploration of TWSL_1’s bioremediation potential.

## Introduction

Industrialization has brought tremendous progress and economic growth, but it has also brought significant environmental challenges. Heavy metal pollution has emerged as a critical global concern due to its harmful impact on ecosystems and human health. Textile dyeing industries, in particular, are recognized as major contributors to heavy metal contamination, releasing effluents laden with toxic metals such as Cd, Zn and Pb into water bodies. The unabated discharge of these pollutants necessitates urgent action to identify and develop efficient remediation strategies. Micro-organisms, particularly bacteria, have emerged as promising candidates for bioremediation due to their ability to tolerate, accumulate and detoxify heavy metals from contaminated environments. Various bacterial species have been studied for their metal resistance and bioremediation potential. Notable examples include *Pseudomonas*, *Bacillus*, *Cupriavidus* and *Streptomyces* species, all of which have demonstrated mechanisms for metal sequestration and detoxification [1,3]. Bacterial metal resistance mechanisms are a foundation for devising eco-friendly approaches to combat heavy metal pollution.

In this context, *Staphylococcus warneri*, a Gram-positive bacterium, has shown significant promise due to its capacity to thrive in harsh industrial effluent environments [4,6]. Notably, the resistance of bacteria to heavy metals is often linked to specific genetic determinants that code for various metal-binding and detoxification proteins, which function through various mechanisms, including metal efflux, sequestration, enzymatic detoxification and intracellular compartmentalization [7,8]. Efflux pumps such as CadA, a cadmium-transporting ATPase, play a crucial role in cadmium resistance by actively exporting Cd²^+^ from bacterial cells, as observed in *Staphylococcus aureus* strain 135. Similarly, the CzcCBA efflux system in *Ralstonia metallidurans* effectively removes cadmium (Cd²^+^), zinc (Zn²^+^) and cobalt (Co²^+^) from the cytoplasm, preventing toxicity. In the case of copper resistance, CopA and CopB, found in *Staphylococcus* and *Enterococcus* species, encode P-type ATPases responsible for copper homeostasis and detoxification by pumping Cu^+^ across membranes. Additionally, metal-binding proteins such as metallothioneins, which are cysteine-rich proteins, chelate heavy metals and mitigate toxicity. For example, metallothioneins in *Pseudomonas putida* bind and sequester cadmium and lead, reducing their bioavailability. Enzymatic detoxification also plays a role, as seen with the ArsC arsenate reductase, which reduces arsenate (As⁵^+^) to arsenite (As³^+^) for subsequent extrusion by the ArsB efflux pump, a mechanism extensively studied in *Escherichia coli* R773. Zinc and cobalt resistance mechanisms involve CzcD, a transmembrane transporter that regulates intracellular Zn²^+^ and Co²^+^ concentrations in *S. aureus* and *Pseudomonas aeruginosa* [9]. These protein-encoding genes form a robust defence against metal toxicity, allowing the bacteria to endure hostile conditions.

Given the complexity and diversity of these resistance mechanisms, whole-genome sequencing (WGS) is ideal for elucidating the complete genetic repertoire of metal-tolerant bacteria [10,11]. WGS enables the comprehensive identification of genes involved in heavy metal resistance and facilitates taxonomic characterization of novel environmental isolates. In the present study, we employed WGS to characterize the genome of *Staphylococcus* sp. TWSL_1, a highly metal-resistant strain isolated from the effluent of a textile dyeing industrial plant. This genome-wide analysis enabled us to investigate genes responsible for metal detoxification and resistance while supporting the accurate taxonomic placement of the isolate. Together, these genomic insights into the heavy metal resistance profile and taxonomic identity of strain TWSL_1 offer a valuable contribution to the knowledge of microbial metal tolerance [12,13]. By understanding the molecular mechanisms underlying metal resistance, this work lays the foundation for developing targeted, genome-informed strategies in bioremediation, particularly for monitoring and mitigating heavy metal contamination in industrial wastewater environments.

## Methods

### Sample collection

An effluent sample was collected from a textile dyeing industry in the Western province of Sri Lanka. To minimize surface contamination, samples were taken from a depth of ~3–7 cm. The samples were collected after the chemical treatment process, with multiple sub-samples obtained from different points within the same location to ensure representative sampling.

### Isolation and selection of the heavy metal-resistant bacterium

To screen potential metal-tolerant bacterial strains, 50 µl aliquots from serial dilutions of the effluent (10^−1^ to 10^−6^) were spread onto Luria Bertani agar plates. After 12 h of incubation at 37 °C, distinct bacterial colonies were re-streaked for isolation. The selected isolated strains were tested for heavy metal resistance by evaluating their growth on LB agar plates supplemented with a mixture of Cd²^+^, Pb²^+^ and Cu²^+^, each at a concentration of 20 mg l^−1^, over a 2 day incubation period. The heavy metals introduced into the LB agar plates were prepared using filter sterilized stock solutions of respective metal salts: CdCl₂ (MW=183.32 g mol^−1^) for cadmium, Pb(NO₃)₂ (MW=331.2 g mol^−1^) for lead and CuSO₄ (MW=159.6 g mol^−1^) for copper. Among the isolates, the strain designated TWSL_1 demonstrated promising heavy metal resistance and was selected for further study.

### Heavy metal removal capacity and heavy metal tolerance of the isolated bacterium

An LB broth (100 ml) spiked with heavy metals, including Cd²^+^, Pb²^+^ and Cu²^+^ (each at 1 mg l^−1^), was inoculated with 50 µl of an overnight-grown TWSL_1 culture, containing ~2×10⁷ c.f.u. ml^−1^. Another LB broth served as an abiotic control without any inoculum. The growth of TWSL_1 over time was monitored at regular intervals (1, 3, 5 and 7 days) by measuring the OD at 600 nm (OD_600_) using a scanning UV-VIS spectrophotometer [Spectro UV-VIS Dual Beam (Split), Helios Alpha, Thermo Fisher Scientific]. To determine the MIC of heavy metals, 10 µl of an overnight-grown TWSL_1 culture was spread on LB agar plates supplemented with various concentrations of metal ions (ranging from 1 to 1,000 mg l^−1^). The MIC was defined as the concentration at which no growth was observed on the third day after incubation. To assess the heavy metal removal capacity of the strain, the concentrations of metals (Cd²^+^, Pb²^+^ and Cu²^+^) in the cultures were determined at the same predefined time intervals using an atomic absorption spectrophotometer [GBC 932 Plus]. Before analysis, aliquots from the samples were filter sterilized (0.25 µm) to remove any living cells. The bioremoval percentage (BR%) of metal ions was calculated using equation 1, where *C*_0_ represents the metal ion concentration at time 0 min and *C*_*t*_ represents the concentration at the specified time interval (*t*) [14,15].


(1) $$\text{BR}\%=[(C_{0}-C_{t})/C_{0}]\times 100.$$

The data were statistically analysed using GraphPad Prism 8.1.1 (330) statistics software.

In addition to the calculation of the mean and sd of sample replicates (*n*=3), one-way ANOVA and two-way ANOVA were used to compare and understand significant differences in growth and bioremoval of metal ion concentrations within the group and between groups. The test was performed at 95% CIs, and the relationship where *P*<0.05 was considered statistically significant. The graphs were plotted, and error bars indicate ±sd.

### Phenotypic and biochemical characterization of the strain

The colony and cellular morphology of pure colonies of TWSL_1 were observed using a compound light microscope (Olympus CKX41), and additional tests, including Gram staining and motility assessment, were conducted according to Benson’s manual [16]. Biochemical tests, such as the methyl red test, starch hydrolysis test, urease test, catalase test, Simmons citrate agar test, coagulase test, gelatin hydrolysis test and MacConkey agar test, were also carried out [16].

### Strain identification

The strain TWSL_1 was molecularly characterized based on the sequence of the 16S rRNA gene. Genomic DNA was initially extracted using the optimized guanidine thiocyanate-based method [15,17]. Subsequently, the 16S rRNA gene was PCR-amplified using the universal primer pair 27F (5′-AGAGTTTGATCCTGGCTCAG-3′) and 1492R (5′-TACGGYTACCTTGTTACGACTT-3′), each at a final concentration of 0.5 µM, with GoTaq DNA polymerase of 1U (Promega), dNTPs at 200 µM, 50 ng of DNA and universal PCR buffer with MgCl_2_ (1.5 mM) [18]. The PCR reaction was performed with initial denaturation at 98 °C for 3 min, followed by 35 cycles of denaturation at 98 °C for 30 s, annealing at 59 °C for 30 s and extension at 72 °C for 90 s, with a final extension at 72 °C for 5 min. The amplified PCR product was purified and subjected to DNA sequencing (Macrogen, Republic of Korea) to elucidate the genetic identity. The complete 16S rRNA sequence of the TWSL_1 strain was compiled using Snap Gene (v. 7.1.2) and submitted to the GenBank repository. The 16S rRNA gene sequence of strain TWSL_1 was aligned with those of closely related taxa retrieved from the National Center for Biotechnology Information (NCBI) GenBank database using the MAFFT online alignment server. Phylogenetic reconstruction was conducted using IQ-TREE version 2.4.0, employing the maximum likelihood method with the HKY+F+I+R2 substitution model, which was identified as the best-fit model by ModelFinder based on the Bayesian information criterion. Node support was assessed using 1,000 ultrafast bootstrap replicates. The resulting phylogenetic tree was visualized using FigTree software (v 1.4.4).

### WGS and assembly

WGS was performed for the extracted genomic DNA of the TWSL_1 strain using the Illumina sequencing platform [19]. The sequencing library was prepared with the Nextera XT DNA Library Preparation Kit (96 samples), followed by cluster generation through bridge amplification. Each cluster was sequenced using Illumina’s sequencing by synthesis technology. Post-sequencing, base identification and quality prediction were performed, and the raw data were analysed using FastQC (v0.11.5). Filtered data statistics were obtained using Trimmomatic (v0.36) to remove adapter sequences and low-quality reads with a quality threshold of Q20 [20]. Overall data quality was assessed with base quality plots generated by FastQC. Then, the filtered reads were *de novo* assembled using the SPAdes genome assembler (v3.15.0), generating an initial draft assembly. To improve genome continuity and accuracy, the assembly was subjected to manual curation and reference-guided scaffolding using the *S. warneri* genome (GCF_018401035.1) to resolve misassemblies and close potential gaps. Error correction was performed subsequently using Pilon v1.24 to enhance base-level accuracy and refine the consensus sequence. Genome circularization was confirmed by identifying a single, complete contig with overlapping terminal sequences, indicative of a closed circular chromosome. Assembly quality and completeness were further evaluated through self-mapping analysis, including average sequencing depth assessments, genome coverage and insert size distribution. Genome coverage was calculated as the number of bases generated divided by the estimated genome. Following draft genome assembly, each scaffold was analysed using blastn against the NCBI nucleotide database to determine its closest taxonomic match [21,22]. The completeness of the genome assembly was evaluated using Benchmarking Universal Single-Copy Orthologs (BUSCO) analysis (v3.0.2) [23].

### Identification of the genome features of the strain TWSL_1

To determine the genomic characteristics of TWSL_1, both average nucleotide identity (ANI) and digital DNA–DNA hybridization (dDDH) analyses were performed using reference genome sequences of the same taxon (NCBI: txid1292) available in the NCBI GenBank database. ANI calculations were obtained using the orthologous average nucleotide identity tool (OAT) with USEARCH [24]. The dDDH calculations employed the genome to genome distance calculator version 3.0 (https://ggdc.dsmz.de/ggdc.php#) [25]. Functional elements, including CDSs, rRNA, tRNA and non-coding RNA, were predicted using Prodigal, RNAmmer, Aragon and Infernal software, respectively. Genome annotation was performed using the integrative analysis pipeline Prokka (v1.14.6) [26]. Following the prediction of CDSs, functional annotation was conducted by matching the predicted protein sequences to the KEGG database via Rapid Annotation using Subsystem Technology (RAST). Specifically, the genome search through RAST was utilized to investigate predicted genes involved in heavy metal resistance [27,30].

While 16S rRNA phylogeny is commonly used for bacterial classification, it lacks the resolution needed to distinguish closely related strains and detect strain-specific adaptations. Therefore, WGS-based phylogenetic analysis was conducted to provide a higher-resolution evolutionary framework and a more comprehensive comparison of genetic divergence. Closest reference genomes were identified using Mash/MinHash, and conserved protein-coding genes were selected from PATRIC global protein families (PGFams). Protein sequences were aligned using muscle, and corresponding nucleotide sequences were mapped to ensure consistency. The tree is built on a concatenated alignment of 120 conserved proteins using maximum likelihood. The final concatenated dataset was analysed using RAxML with the GTR+GAMMA substitution model, and fast bootstrapping was applied to assess tree reliability. The phylogenetic tree was visualized with branch support values, with the scale bar representing evolutionary distance in nucleotide substitutions per site. The complete analysis was done using BV-BRC web resources (https://www.bv-brc.org/).

To further highlight the genes involved in heavy metal resistance in strain TWSL_1, a circular genome map was created using the Proksee server, incorporating annotation data from Prokka [26]. The complete assembled draft genome was deposited in the NCBI GenBank repository.

## Results and discussion

### Isolation and evaluation of the heavy metal resistance strain

**Fig. 1.**
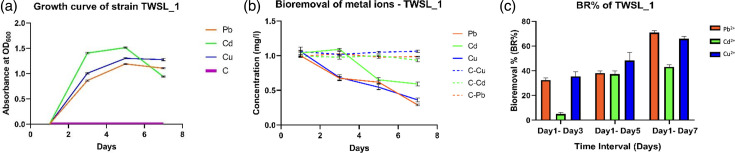
Metal removal capability and tolerance of the bacterial strain TWSL_1 over 7 days. (**a**) Growth of the TWSL_1 strain from day 1 to day 7 in cultures spiked with different metal ions (Cd²^+^, Pb²^+^ and Cu²^+^) (mg l^−1^). ‘C’ represents the abiotic control, which contains LB broth with metals but without bacterial inoculum. (**b**) Heavy metal removal by TWSL_1 over time. (**c**) Percentage of metal removal over time. The assay utilized media enriched with Cu²^+^, Cd²^+^ and Pb²^+^ (1 mg l^−1^), with corresponding abiotic controls labelled as C-Cu, C-Cd and C-Pb. Error bars indicate sd (*n*=3). The significance of mean differences was set at *P*≥0.05.

From the pool of bacterial strains cultivated on LB agar plates, ten isolates were obtained as pure colonies. Among these, a strain exhibiting robust growth in a multi-metal-enriched medium was selected and designated as TWSL_1. This strain demonstrated resistance to Cd²^+^, Pb²^+^ and Cu²^+^, showing significant growth (*P*<0.0001) in metal-spiked media. Notably, TWSL_1’s growth in the presence of these metal ions differed significantly (*P*<0.05), maintaining steady growth for up to 5 days (Fig. 1a). Initially, the highest growth rate was observed with Cd²^+^, while the lowest was with Pb²^+^. By the seventh day, Cu²^+^ induced a higher growth rate compared to Cd²^+^ and Pb²^+^. This variation in growth rates likely reflects the different detoxification and resistance mechanisms activated by the bacterium in response to each metal ion. The observed initial stress-induced drop in growth rate, followed by a recovery phase, underscores the activation of detoxification pathways. Such behaviour is indicative of an adaptive response where TWSL_1 mobilizes its defence mechanisms to mitigate metal toxicity, thereby resuming growth. The continuous growth of TWSL_1 throughout the experiment demonstrates its high tolerance for heavy metals, a trait that is beneficial for bioremediation applications. Similar findings have been reported in other studies where bacterial strains adapted to heavy metal stress exhibited initial growth inhibition, followed by recovery, highlighting their detoxification capabilities [31]. TWSL_1 showed high resistance to Pb²^+^ with an MIC of 1,200 mg l^−1^, while the MIC values for Cd²^+^ and Cu²^+^ were 50 and 75 mg l^−1^, respectively. These MIC values are consistent with those observed in other metal-resistant bacterial strains, such as *S. aureus*, where resistance to Pb²^+^ often exceeds that of Cd²^+^ and Cu²^+^ due to different metal uptake and efflux systems [32].

Moreover, TWSL_1 demonstrated significant metal removal abilities over 7 days, with the strain effectively reducing metal concentrations compared to the control (*P*<0.05, *P*<0.0001) (Fig. 1b, c). The control samples showed no significant reduction in metal ion concentrations (*P*<0.01), confirming that the decrease was solely due to the metal removal capability of TWSL_1. By the seventh day, TWSL_1 removed over 40% of the tested metal ions from their initial concentrations (1 mg l^−1^) (*P*<0.05) (Fig. 1c). The highest BR% on the seventh day were 43.07±1.70% for Cd²^+^, 70.98±1.41% for Pb²^+^ and 65.85±1.85% for Cu²^+^. These significant bioremoval efficiencies strengthen TWSL_1’s potential in bioremediation applications. These findings suggest that TWSL_1’s heavy metal removal mechanisms are activated upon metal exposure.

These findings are supported by several studies demonstrating similar metal removal capabilities in other bacterial strains. For instance, a study by Khan *et al*. [33] on a *Bacillus* strain isolated from industrial wastewater showed significant removal efficiencies for Cd²^+^, Pb²^+^ and Cu²^+^, highlighting the role of microbial biomass in biosorption and bioaccumulation processes [33]. Similarly, Wang *et al*. [34] demonstrated that a *Pseudomonas* strain could remove heavy metals through biosorption, with the highest removal rates for Pb²^+^ and Cu²^+^, aligning with the efficiencies observed for TWSL_1 [34]. Furthermore, the findings of Das *et al*. [35] on the heavy metal removal efficiency of a *Streptomyces* strain are comparable to those of TWSL_1, particularly for Pb²^+^ and Cu²^+^[35]. Gadd and Griffith [36] had disclosed the role of microbial metabolism in metal detoxification, emphasizing how certain bacteria, including *Staphylococcus* species, employ metallothionein and other proteins to bind and sequester heavy metals, thereby reducing their bioavailability and toxicity [36].

### Phenotypic and biochemical characterization of the strain

Microscopic examination of the TWSL_1 strain revealed non-motile, Gram-positive cocci arranged in irregular, grape-like clusters, a hallmark of *Staphylococcus* species [37]. The colonies exhibited an off-white, circular appearance with marginated, raised and shiny/wet surfaces, consistent with typical *Staphylococcus* morphology. Biochemical assays further supported this classification, showing positive results for starch hydrolysis, urease and catalase [37]. These characteristics are important for distinguishing *Staphylococcus* from other Gram-positive cocci, particularly due to the catalase test, differentiating it from catalase-negative *Streptococcus* species [38,39]. The ability to hydrolyse starch and produce urease are additional phenotypic traits frequently observed in *Staphylococcus* species [40]. Therefore, the microscopic and biochemical profiles of TWSL_1 classify it within the *Staphylococcus* genus.

### Strain characterization and 16S rRNA gene phylogeny of *TWSL_1*

**Fig. 2.**
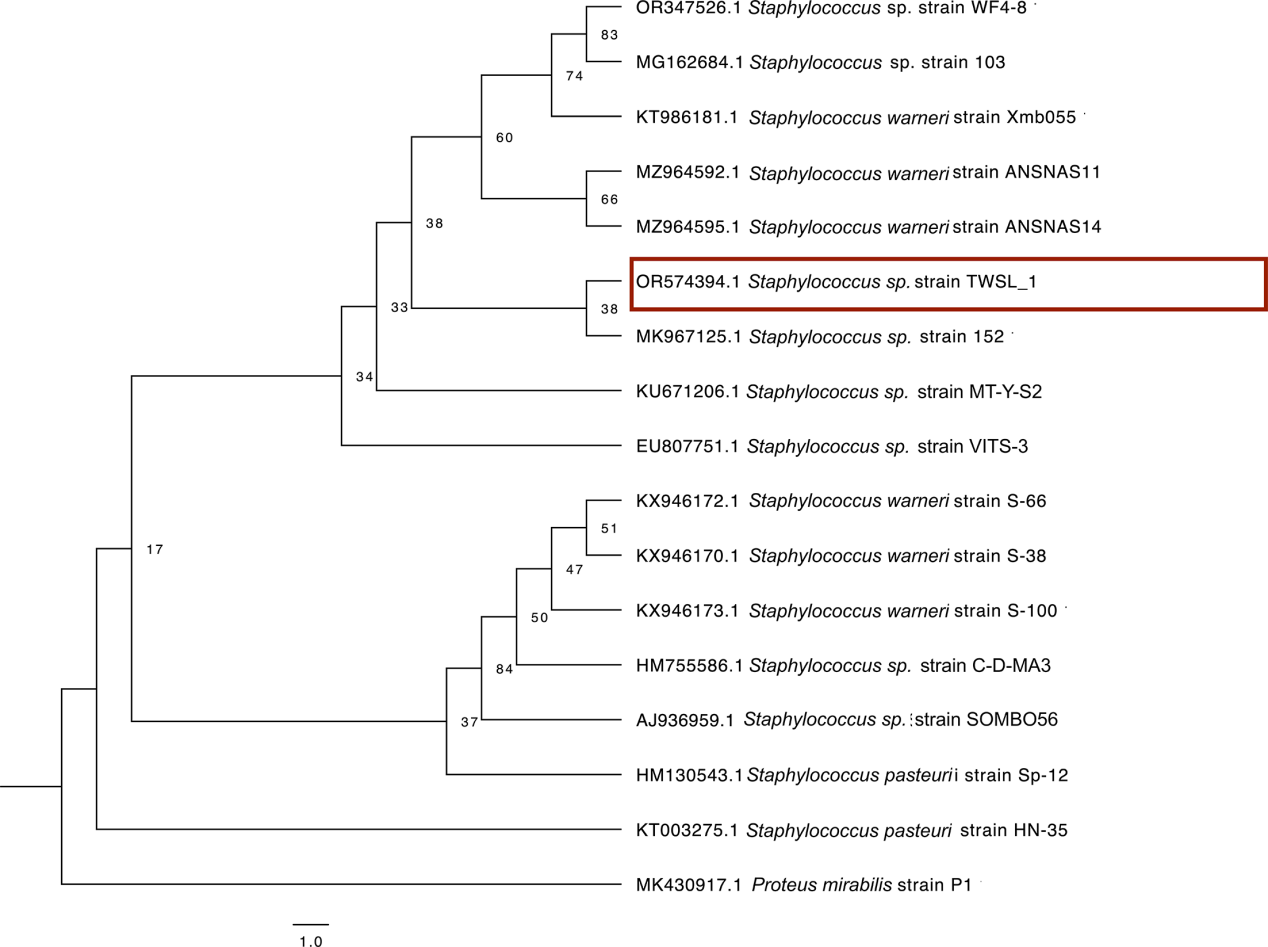
Maximum likelihood phylogenetic tree based on the 16S rRNA gene sequence of strain TWSL_1 and closely related species within the genus *Staphylococcus*. The tree was constructed using IQ-TREE and visualized with FigTree (v1.4.4). GenBank accession numbers are indicated at the beginning of each taxon label. Bootstrap percentages (≥50%) based on 1,000 replications are given at branch nodes. *Proteus mirabilis* P1 was used as an outgroup.

blast analysis of the PCR-amplified 16S rRNA sequence (1,299 bp) revealed a 98% similarity to the 16S rRNA sequence of * S. warneri* strain ANSNAS11 (accession no.: MZ964592.1) in the NCBI GenBank. Phylogenetic analysis based on the 16S rRNA sequences (Fig. 2) illustrated the genetic relationship between *Staphylococcus* strain TWSL_1 and other *Staphylococcus* strains.

Phylogenetic analysis based on the 16S rRNA gene sequence revealed that the strain TWSL_1 (highlighted with a red rectangle shape) clustered closely with members of the genus *Staphylococcu*s, with the highest similarity to *S. warneri*. The maximum likelihood tree (Fig. 2) showed that TWSL_1 grouped within a well-supported clade containing *S. warneri* reference strains, supported by high bootstrap values (>90%). This phylogenetic placement supports the taxonomic identification of the isolate as *S. warneri* and is consistent with results obtained through blast-based similarity searches. *Proteus mirabilis* P1 has been included in these analyses as an outgroup to root the phylogenetic trees; it provides a reference point that will help in distinguishing *Staphylococcus* lineages from other bacterial groups. Therefore, the strain was confirmed to belong to the *Staphylococcus* genus, and its 16S rDNA sequence was deposited in the NCBI repository (accession no.: OR574394.1).

### Genome features of the strain *Staphylococcus* strain TWSL_1

Accurate species-level classification is essential for understanding the ecological roles and biotechnological potential of environmental bacterial isolates. Traditionally, 16S rRNA gene sequencing has been widely used for bacterial taxonomy due to its conserved and variable regions that facilitate phylogenetic inference. However, while effective at the genus level, this method often lacks the resolution needed to distinguish between closely related species, particularly within genera like *Staphylococcus*, where interspecies similarity frequently exceeds 98.5% [41,42]. To overcome this limitation, whole-genome-based approaches have emerged as the gold standard for bacterial species delineation. Therefore, a combination of genomic tools, including ANI, OrthoANI, dDDH and core-genome phylogenetic analysis, was employed in this study, as each offers distinct strengths in resolving taxonomic boundaries and elucidating evolutionary relationships. These genome-based methods enabled robust species-level identification and provided a foundation for downstream genomic comparisons. Specifically for strain TWSL_1, this integrated approach confirmed its classification as *S. warneri* clarified its phylogenetic placement and facilitated the identification of key genes associated with heavy metal resistance.

### Genome statistics

The assembled draft genome of *Staphylococcus* strain TWSL_1 was deposited in the NCBI GenBank under accession no. CP135051. The genome of the strain TWSL_1 was characterized by the total genome length of 2,661,318 bp, with 2,603 predicted CDSs. Moreover, the genome analysis revealed the presence of 59 tRNAs, 8 rRNAs and 1 tmRNA. Importantly, the genomic DNA G+C content was determined to be 33 mol%, falling within the reported range for the *Staphylococcus* genus (30–40%), as previously documented in various genomic studies [43,46]. A summary of the genome sequencing project information is given in Table 1.

**Table 1.** Genome assembly characteristics

Attribute		Value
Genome size		2.7 Mb
G+C content (mol%)		32.8
Total genes		2,635
Protein-coding genes		2,603
RNA genes		68
L50		1
Genome coverage		487×
BUSCO analysis	Complete and single-copy BUSCOs	124 (100%)
	Complete and duplicated BUSCOs	0 (0%)
	Fragmented BUSCOs	0 (0%)
	Missing BUSCOs	0 (0%)

### Average nucleotide identity

**Fig. 3.**
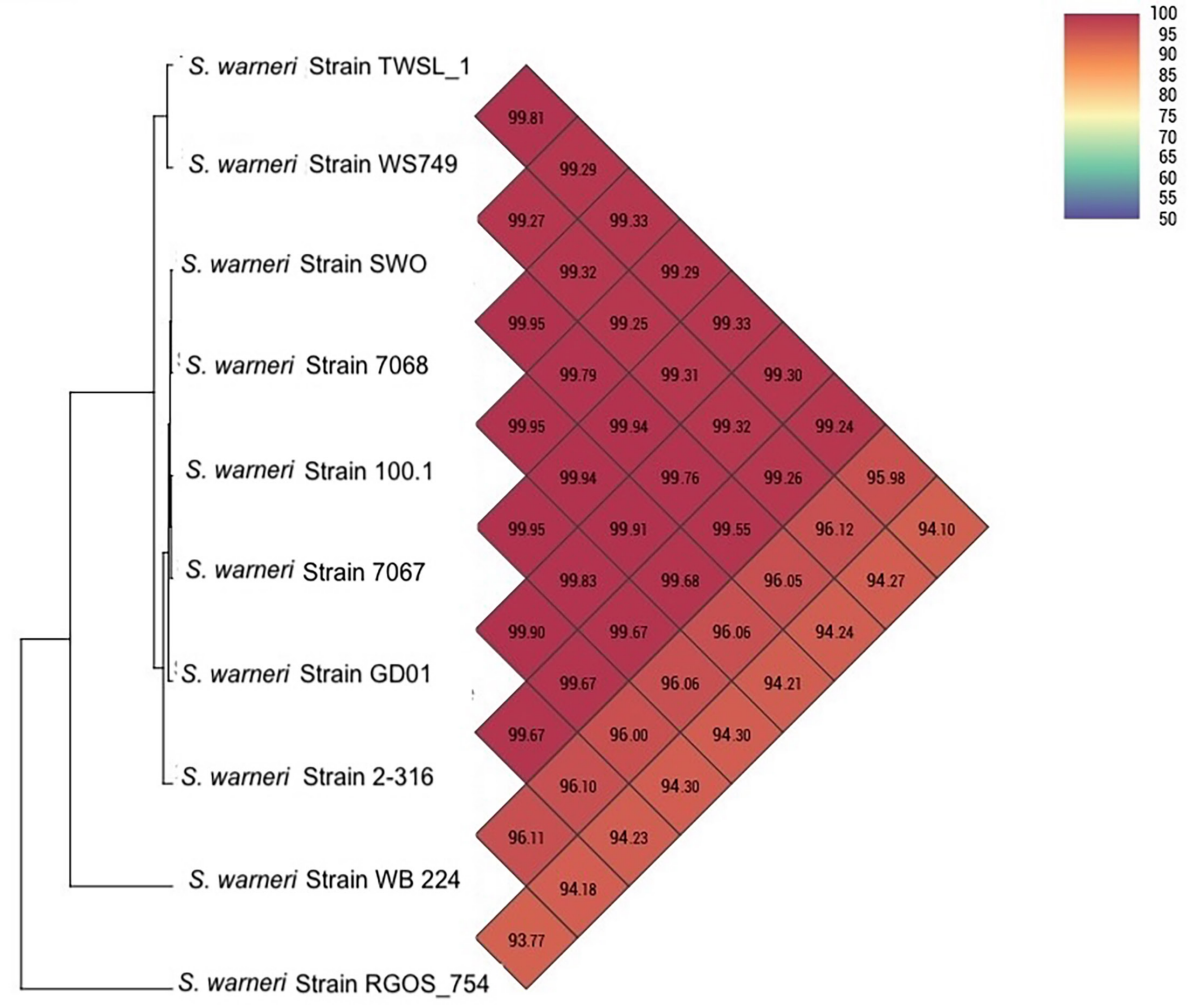
Heatmap of ANI values generated using OrthoANI software, comparing the complete genome sequence of the tested strain TWSL_1 with various *S. warneri* strain genomes. The colour gradient represents the ANI values, with darker red indicating higher similarity.

The ANI heatmap was generated using OrthoANI values (Fig. 3) from the OAT software. OrthoANI is a refinement of ANI that focuses on orthologous gene pairs and further improves species-level resolution [24]. The results indicated a high level of genomic similarity between TWSL_1 and other *S. warneri* strains, with values ranging from 99.27 to 99.81%. These results confirm that TWSL_1 is closely related to these strains, clearly placing it within the *S. warneri* species. Such high ANI values, typically above 95.1%, are consistent with species-level classification supporting the identification of TWSL_1 as a strain of *S. warneri*.

**Fig. 4.**
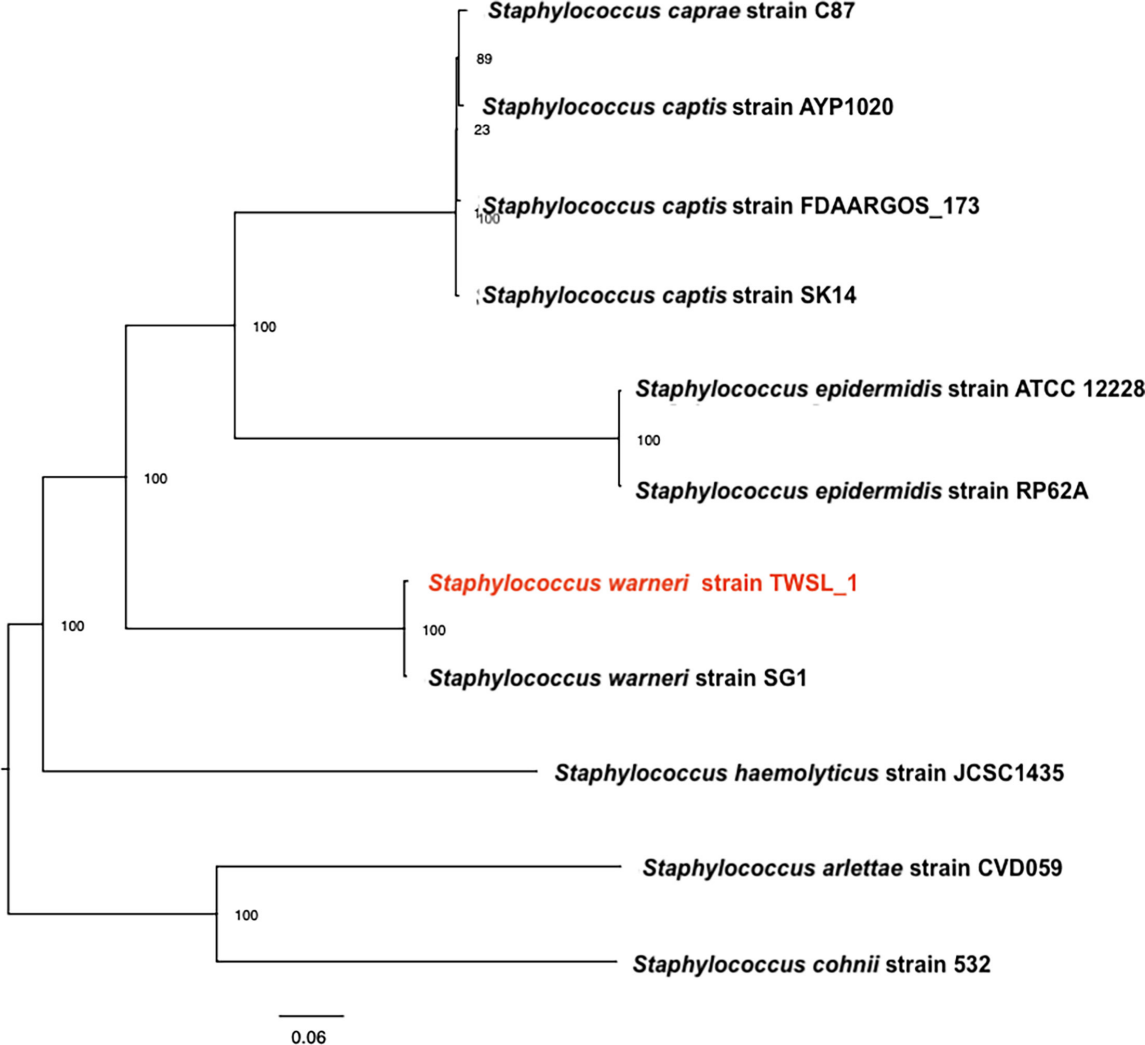
Phylogenetic tree of *S. warneri* strain TWSL_1 and its closest reference genomes, inferred using a whole-genome sequence-based approach (https://www.bv-brc.org/).

The TWSL_1 strain shows the highest ANI value (99.81%) with *S. warneri* WS479 (accession no.: CP061041.1), suggesting a particularly close genomic relationship with this strain. Compared to other closely related strains, including SWO, 7068, 7067 and GD01 (accession no.: NZ_CP033098.1, CP118789.1, CP118794.1 and NZ_CP038242.1), TWSL_1 maintains ANI values above 99%, further reinforcing its placement within the same species cluster. Furthermore, dDDH emulates traditional wet-lab DDH techniques through *in silico* genome comparisons. It provides another quantitative framework for species delineation, with a 70% similarity threshold representing the classical species cutoff, and offers complementary validation to ANI/OrthoANI, especially in cases where ANI values are borderline [24,25]. The dDDH value between *Staphylococcus* sp. TWSL_1 and the reference *S. warneri* type strains was>87%, exceeding the 70% threshold for species delineation. This genomic proximity highlights the robustness of ANI and dDDH as metrics for species identification and delineation in microbial genomics, demonstrating that TWSL_1 shares significant genetic continuity with established * S. warneri* strains while maintaining some distinguishable features from more divergent relatives. Therefore, the *Staphylococcus* strain TWSL_1 was confirmed as *S. warneri* TWSL_1 (Fig. 4).

### Whole-genome-based phylogeny

Beyond pairwise similarity metrics, the core-genome phylogenetic analysis offers a high-resolution approach to understanding evolutionary relationships by comparing conserved genes across multiple genomes and involves identifying the shared gene set (core genome) among all strains, aligning those sequences, and constructing a phylogenetic tree based on concatenated alignments [47,48]. The core-genome phylogenetic analysis demonstrated that *S. warneri* strain TWSL_1 clusters closely with *S. warneri* SG1, forming a distinct clade with a strong bootstrap value of 100, indicating high confidence in their evolutionary relationship. This robust clustering suggests a significant genomic similarity between TWSL_1 and SG1, potentially reflecting shared genetic traits and functional characteristics. The *S. warneri* clade is well separated from other *Staphylococcus* species, including *Staphylococcus epidermidis*, which also forms a strongly supported clade (bootstrap value 100) but remains distinct from *S. warneri*. Similarly, the phylogenetic structure supports the divergence of *Staphylococcus capitis*, *Staphylococcus caprae*, *Staphylococcus haemolyticus* and *Staphylococcus arlettae*, with high bootstrap values confirming the reliability of these branch points. The consistent bootstrap support across the tree reinforces the classification of TWSL_1 within *S. warneri* while highlighting its genetic proximity to SG1 rather than other *Staphylococcus* species. These findings provide strong evidence for the evolutionary placement of strain TWSL_1, suggesting it shares a common ancestor with SG1 yet remains distinct from other members of the genus.

### Subsystem classification

**Fig. 5.**
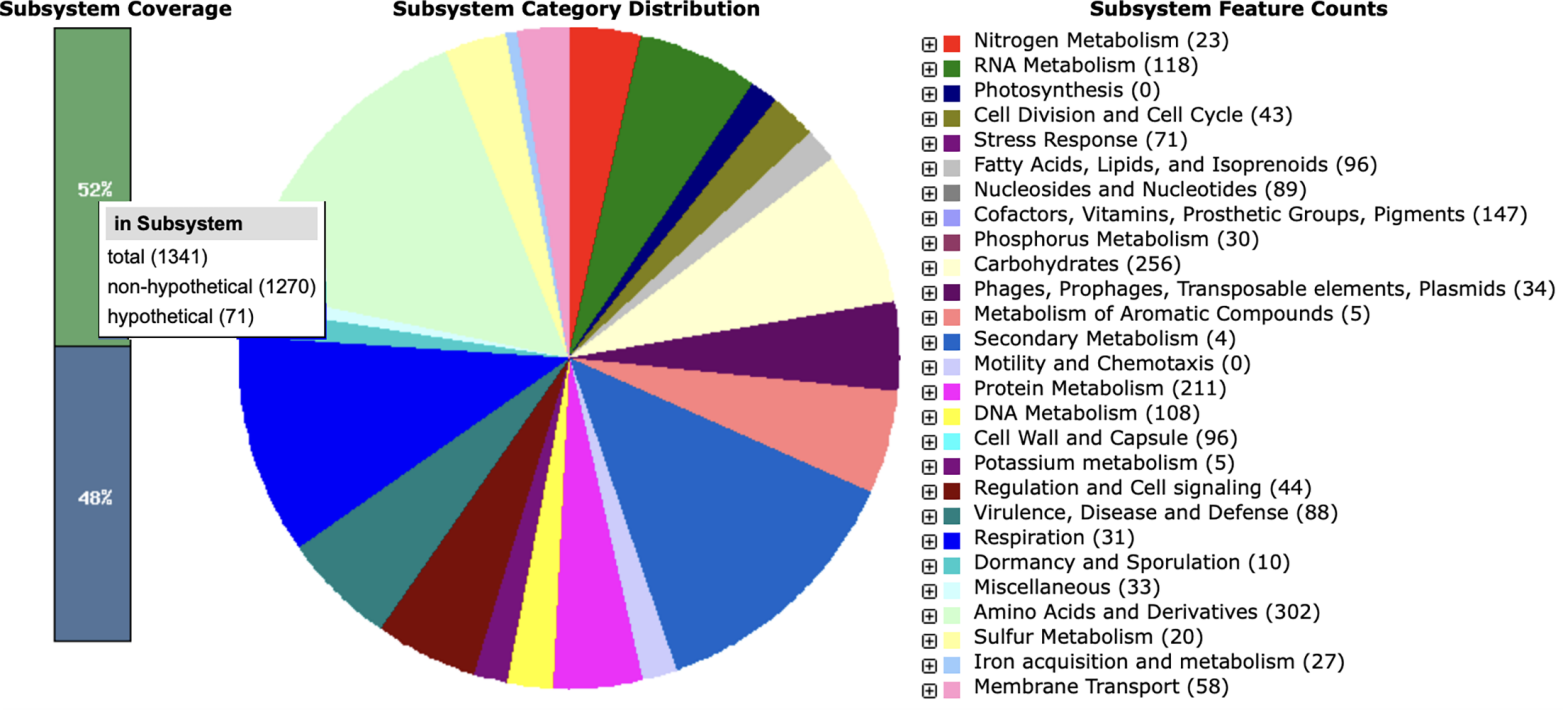
Subsystem classification of *S. warneri* strain TWSL_1 as identified through RAST annotation. (Data are presented in two main visual elements: a bar chart illustrating subsystem coverage and a pie chart displaying the distribution of subsystem categories along with their feature counts.)

The subsystem distribution for the *S. warneri* strain TWSL_1 revealed a broad spectrum of functional gene categories, with notable prevalence in several key areas (Fig. 5).

The subsystem distribution analysis provides valuable insights into the metabolic and functional capabilities of *S. warneri* strain TWSL_1. The significant representation of amino acid and carbohydrate metabolism suggests a versatile and adaptive metabolic profile, enabling the organism to thrive in diverse environments. Amino acids, their derivatives such as polyamines, peptides and specialized cofactors, and carbohydrates are critical for various cellular processes, including energy production, structural integrity and biosynthesis of essential molecules. Numerous virulence and defence-related features support potential pathogenic traits, which may have implications for understanding *S. warneri* TWSL_1’s interactions with hosts and its role in microbial communities. These traits can help the bacterium evade the host immune system, produce toxins or resist antimicrobial agents. Identifying these features aligns with findings in other strains of *S. warneri*, known to possess mechanisms for pathogenicity and resistance to environmental stressors. In previous studies, *S. warneri* has been documented to exhibit a diverse range of metabolic capabilities, including the ability to metabolize various amino acids and carbohydrates, which supports its survival in different ecological niches [49,50]. The genetic and translational efficiency, indicated by the features related to protein, RNA and DNA metabolism, underscores its adaptability and robustness.

### Comparative genomic analysis of heavy metal resistance

The comparative genomic analysis of *S. warneri* strain TWSL_1 was conducted to investigate its heavy metal resistance mechanisms relative to other *Staphylococcus* species. Using the RAST server, *S. warneri* TWSL_1 was compared with * S. epidermidis* ATCC 12228 and *S. aureus* RF122 to identify genetic determinants common to environmental *Staphylococcus* species but absent in pathogenic strains. This analysis provided insights into the evolutionary divergence of metal resistance mechanisms within the genus and highlighted the potential of *S. warneri* TWSL_1 for bioremediation. The results revealed that *S. warneri* TWSL_1 harbours an extensive array of genes associated with heavy metal resistance, emphasizing its adaptive capacity in metal-contaminated environments. Notably, it possesses genes such as the cadmium efflux system accessory protein (22,286,620–22,286,967 bp) and the cadmium resistance protein (2,285,984–2,286,601 bp), both vital for cadmium detoxification. These genes are absent in *S. aureus* RF122, suggesting a unique adaptation in *S. warneri* TWSL_1. Additionally, the presence of cobalt-zinc-cadmium resistance genes (*czcD* proteins at 2,256,527–2,255,586 bp and 2,219,839–2,220,783 bp), along with a Zn (II) and Co (II) transmembrane diffusion facilitator (2,418,672–2,417,725 bp), indicates a robust resistance mechanism against multiple heavy metals. The strain also harbours the copper-translocating P-type ATPase gene (2,266,896–2,264,833 bp) and copper resistance protein D (1,081,428–1,080,175 bp), reinforcing its ability to maintain copper homeostasis, which is notably absent in *S. aureus* RF122. These findings underscore the genetic resilience of *S. warneri* TWSL_1 under heavy metal stress, demonstrating its capability to thrive in contaminated environments.

**Fig. 6.**
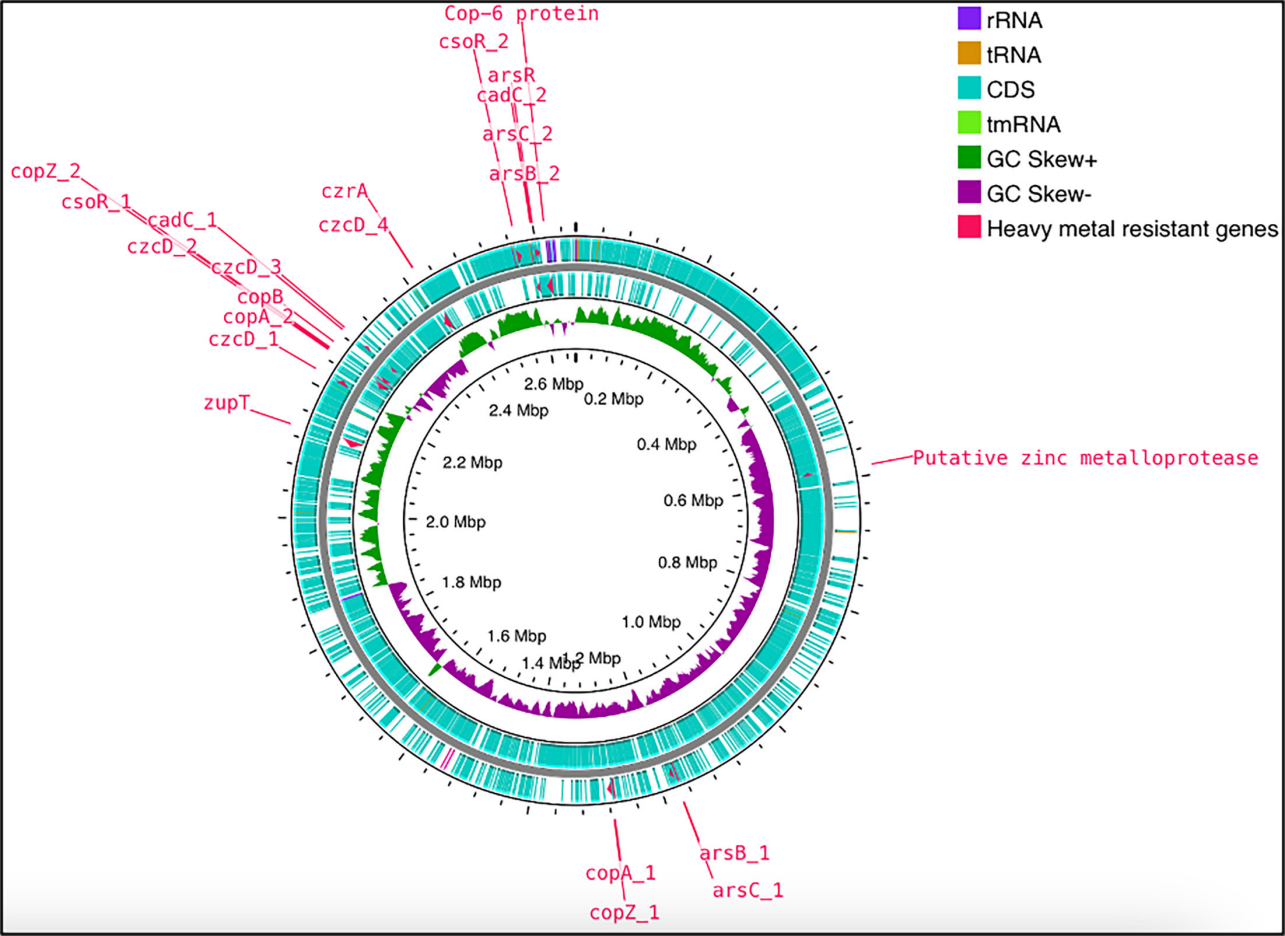
Circular representation of the complete genome and heavy metal-resistant protein-coding genes of *S. warneri* TWSL_1. The genome map is organized from the outermost to the innermost circles. The second outermost ring shows the annotated CDSs of strain TWSL_1. Predicted heavy metal resistance genes are highlighted in red. These annotations were derived from the *de novo* assembled and annotated genome of TWSL_1.

To further investigate strain-specific adaptations, a comparative genome analysis was performed between *S. warneri* TWSL_1 and *S. warneri* strain 22.1. This intraspecies comparison minimized phylogenetic variations, allowing for a precise evaluation of unique resistance traits. Both strains share key metal resistance genes, including those encoding cobalt-zinc-cadmium resistance proteins, copper-translocating P-type ATPases, CopC, CopD, Zn (II) and Co (II) transmembrane diffusion facilitators and the CsoR repressor of the copZA operon, indicating a conserved resistance mechanism within the species. However, *S. warneri* TWSL_1 uniquely harbours the cadmium efflux system accessory protein, a key determinant in cadmium detoxification, which enhances its ability to actively export cadmium ions, reducing cellular toxicity and improving survival in cadmium-contaminated environments. These findings emphasize the genomic advantages of *S. warneri* TWSL_1, distinguishing it as a superior candidate for heavy metal bioremediation. Its expanded resistance repertoire suggests strong adaptability to metal-contaminated environments, making it an ideal strain for further exploration in environmental remediation efforts. This was further supported by the presence of several genomic features and genes related to heavy metal resistance in the circular genome map of *S. warneri* strain TWSL_1 (Fig. 6).

The outer rings display annotated CDSs, rRNA, tRNA and hypothetical proteins. The G+C content and G+C skew are also shown, providing insights into genome stability and replication dynamics. The genes involved in heavy metal resistance are highlighted in red, including *copA* and *copB* (copper-transporting ATPases), *cadC* and *cadD* (cadmium resistance operon components), *csoR* (copper-sensitive operon repressor), *arsR* (arsenic resistance regulator) and *zupT* (zinc uptake transporter). The presence of these genes suggests that *S. warneri* TWSL_1 possesses robust mechanisms for detoxifying and resisting various heavy metals, demonstrating its potential for bioremediation applications [51,55]. These resistance traits are consistent with those found in other *Staphylococcus* species known for similar adaptive responses [30,31].

In conclusion, the genomic characterization of *S. warneri* strain TWSL_1 reveals its remarkable resistance to cadmium, lead and copper, highlighting its adaptation to heavy metal-contaminated environments. WGS and comparative analysis identified key genetic determinants associated with metal resistance, including genes encoding cadmium efflux systems, copper-translocating ATPases and cobalt-zinc-cadmium resistance proteins. These findings underscore the strain’s robust genomic adaptations to metal stress, reinforcing its potential as a promising candidate for bioremediation applications. Future research should focus on the functional validation of these resistance genes and their role in real-world bioremediation settings. Exploring potential genetic modifications or microbial consortia approaches could further enhance the efficiency of *S. warneri* TWSL_1 in environmental clean-up efforts. These insights contribute to leveraging microbial solutions for mitigating heavy metal pollution and advancing sustainable ecological management practices.

## Supplementary material

10.1099/acmi.0.000954.v5 Supplementary Material 1.

## Abbreviations

ANI: average nucleotide identity
BR%: bioremoval percentage
BUSCO: Benchmarking Universal Single-Copy Orthologs
dDDH: digital DNA–DNA hybridization
LB: Luria Bertani
NCBI: National Center for Biotechnology Information
OAT: orthologous average nucleotide identity tool
RAST: rapid annotation using subsystem technology
WGS: whole-genome sequencing
