# Supplementary material for: Multi-metal-resistant Staphylococcus warneri strain TWSL_1: revealing heavy metal-resistant genomic features by whole-genome sequencing and analysis
Source: Access Microbiol. 2025 May 27;7(5):000954.v5. doi: 10.1099/acmi.0.000954.v5 (PMC12117009; doi:10.1099/acmi.0.000954.v5)
Supplement: Supplementary Material 1. [file acmi-7-00954-s001.pdf]

Accession numbers of the data sets used in the study.

Complete genome analysis

CP053477.1 - *Staphylococcus warneri* strain WB224 chromosome, complete genome

CP038242.1 - *Staphylococcus warneri* strain GD01 chromosome, complete genome

CP033098.1- *Staphylococcus warneri* strain SWO chromosome, complete genome

CP061041.1 -*Staphylococcus warneri* strain WS479 chromosome, complete genome

CP054017.1 -*Staphylococcus warneri* strain FDAARGOS_754 chromosome, complete genome

CP118973.1 -*Staphylococcus warneri* strain DSM 20316 chromosome, complete genome

CP049802.1 -*Staphylococcus warneri* strain Cap 100.1 chromosome, complete genome

CP118789.1 -*Staphylococcus warneri* strain 7068 chromosome, complete genome

CP118794.1 -*Staphylococcus warneri* strain 7067 chromosome, complete genome

CP134464.1 -*Staphylococcus warneri* strain 2023.05 chromosome, complete genome

CP032159.1- *Staphylococcus warneri* strain 22.1 chromosome, complete sequence

AJ938182.1- *Staphylococcus aureus* RF122, complete genome

Whole genome phylogenetic analysis

LATV00000000.1-*Staphylococcus cohnii* strain 532, whole genome

CP007601.1- *Staphylococcus capitis* strain AYP1020 chromosome, complete sequence

NZ_WBME00000000.1-*Staphylococcus epidermidis* ATCC 12228, whole genome

NZ_ACFR00000000.1-*Staphylococcus capitis* SK14, whole genome

NZ_VDSH00000000.1-*Staphylococcus warneri* SG1 strain DE0436, whole genome

NZ_LORZ00000000.2-*Staphylococcus capitis* strain FDAARGOS_173, whole genome

NZ_ALWK00000000.1- *Staphylococcus arlettae* CVD059, whole genome

NC_007168.1- *Staphylococcus haemolyticus* JCSC1435, complete genome

NC_002976.3- *Staphylococcus epidermidis* RP62A, complete sequence

NZ_ACRH00000000.1- *Staphylococcus caprae* C87, whole genome

16S rRNA gene analysis

MK430917.1 -*Proteus mirabilis* strain P1 16S ribosomal RNA gene,

AJ936959.1 -*Staphylococcus* sp. SOMBO56 partial 16S rRNA gene

MK967125.1- *Staphylococcus* sp. strain 152 16S ribosomal RNA gene

KT003275.1 -*Staphylococcus pasteuri* strain HN-35 16S ribosomal RNA gene

MZ964592.1- *Staphylococcus warneri* strain ANSNAS11 16S ribosomal RNA gene,

HM755586.1 -*Staphylococcus* sp. C-D-MA3 16S ribosomal RNA gene

MG162684.1- *Staphylococcus* sp. strain 103 16S ribosomal RNA gene,

OR347526.1 -*Staphylococcus* sp. strain WF4-8 16S ribosomal RNA gene

MZ964595.1 -*Staphylococcus warneri* strain ANSNAS14 16S ribosomal RNA gene

EU807751.1 -*Staphylococcus* sp. VITS-3 16S ribosomal RNA gene

HM130543.1 -*Staphylococcus pasteuri* strain Sp-12 16S ribosomal RNA gene

KU671206.1 -*Staphylococcus* sp.MT-Y-S2 16S ribosomal RNA gene

KX946173.1 -*Staphylococcus warneri* strain S-100 16S ribosomal RNA gene

KX946172.1 -*Staphylococcus warneri* strain S-66 16S ribosomal RNA gene

KX946170.1 -*Staphylococcus warneri* strain S-38 16S ribosomal RNA gene

KT986181.1 -*Staphylococcus warneri* strain Xmb055 16S ribosomal RNA gene
